# Osteoporosis in COPD patients: Risk factors and pulmonary rehabilitation

**DOI:** 10.1111/crj.13514

**Published:** 2022-06-10

**Authors:** Yujuan Li, Hongchang Gao, Lei Zhao, Jinrui Wang

**Affiliations:** ^1^ Department of Pulmonary and Critical Care Medicine Shanghai Pudong New Area Gongli Hospital Shanghai China

**Keywords:** chronic obstructive pulmonary disease, fracture, osteoporosis, prevalence, pulmonary rehabilitation, risk factors

## Abstract

**Objectives:**

To present a review on the pathogenesis, risk factor and treatment of chronic obstructive pulmonary disease complicated with osteoporosis and provide new ideas for the diagnosis and treatment.

**Data source:**

A systematic search is carried out using keywords as chronic obstructive pulmonary disease, osteoporosis, risk factors, and pulmonary rehabilitation.

**Results:**

Patients with chronic obstructive pulmonary disease have a high prevalence of osteoporosis and a high risk of fracture. The mechanisms of osteoporosis in COPD patients are associated with general risk factors, such as smoking, reduced physical activity, low weight, and disease‐specific risk factors, such as systemic inflammatory, Vitamin D deficiency, use of glucocorticoid, anemia, hypoxemia, and hypercapnia. The treatment of osteoporosis in COPD emphasizes comprehensive intervention, which mainly include basic treatment and anti‐osteoporosis drugs. Noticeably, pulmonary rehabilitation program is an important part of treatment.

**Conclusions:**

This work summarizes the pathogenesis, risk factor, prevention, and treatment of chronic obstructive pulmonary disease complicated with osteoporosis, and the latest progress of studies on chronic obstructive pulmonary disease and osteoporosis is discussed.

## Graphical abstract

Patients with chronic obstructive pulmonary disease have a high prevalence of osteoporosis and a high risk of fracture. The mechanisms of osteoporosis in COPD patients are associated with general risk factors, such as smoking, reduced physical activity, low weight and disease‐specific risk factors, such as systemic inflammatory, Vitamin D deficiency, use of glucocorticoid, anemia, hypoxemia, and hypercapnia. The treatment of osteoporosis in COPD emphasizes comprehensive intervention. Noticeably, pulmonary rehabilitation program is an important part of treatment.

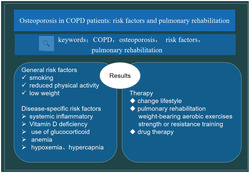

## 1 BACKGROUND

Chronic obstructive pulmonary disease (COPD) is a preventable and treatable condition characterized by progressive, incompletely reversible airflow restriction. According to the epidemiological survey of COPD in China, the prevalence rate of COPD is 8.6% (11.9% for males and 5.4% for females), and the number of patients is nearly 100 million, among which the prevalence rate of COPD in adults over 40 years old is as high as 13.7%.^1^ COPD ranks third in the global cause of death^2^ and fifth in the global economic burden of disease.^3^ In recent years, more and more attention has been paid to its systemic effects,^4^ including cardiovascular and cerebrovascular diseases, metabolic syndrome, osteoporosis, malnutrition, skeletal muscle dysfunction, diabetes, anxiety, depression, and so on. Osteoporosis is a significant extrapulmonary effect in COPD.

Osteoporosis is a systemic bone disease characterized by low bone density and microstructure change that increase the risk of fractures.^5^, ^6^, ^7^, ^8^ Because of the reduction of exercise and long‐term bed, osteoporosis‐related fractures are associated with several adverse health outcomes in COPD, including deteriorated lung function, poor quality of life, increased hospitalization, and mortality rates. Moreover, the two diseases form a vicious cycle and cause significant burden on these patients.

The presence of osteoporosis in patients with COPD is asymptomatic and often undiagnosed until bone fractures occur. Therefore, it is necessary to explore the pathogenesis of osteoporosis in COPD, and special attention should be paid to early recognition of patients at high risk for osteoporosis in COPD.

In this review, we focus on osteoporosis as an extrapulmonary manifestation of COPD. The prevalence, risk factor and potential mechanism of osteoporosis in COPD are discussed and the treatment of osteoporosis is described, especially emphasize on exercise rehabilitation.

## 2 PREVALENCE OF OSTEOPOROSIS IN COPD

A review quantitatively synthesized the current evidence on the prevalence and risk factors for osteoporosis in COPD in 58 studies with 8753 participants with COPD to demonstrate a pooled global prevalence of 38%.^9^ The prevalence of osteoporosis in COPD is 2‐fold to 5‐fold higher than in age‐matched healthy control subjects.^9^, ^10^ A recent study has shown that low volumetric bone mineral density (BMD) is present in 58% of all subjects with COPD, and is even more frequent in those with worse COPD, and has a prevalence of 84% among subjects with very severe COPD.^11^ A meta‐analysis that contained the total number of patients with COPD from all studies is 3815, has shown the prevalence of osteoporosis among COPD is higher than that among healthy subjects (osteoporosis, 14%–66% and osteopenia, 18%–65%).^12^ The difference depends on the diagnostic methods, the population of study, and the severity of the underlying respiratory disease.

## 3 RISK FACTORS AND MECHANISMS FOR OSTEOPOROSIS IN COPD

The mechanisms of osteoporosis in COPD patients are mostly unknown. However, clinical evidence indicates that osteoporosis and other systemic comorbidities of COPD are associated with general risk factors and disease‐specific risk factors. In the following paragraphs, we briefly discuss general risk factors of osteoporosis in COPD patients as well as disease‐associated factors.

### 3.1 General risk factors for osteoporosis in COPD

#### 3.1.1 Smoking

Smoking is a common risk factor for COPD and osteoporosis. Patients with COPD tend to have a long history of smoking. Many studies have shown that smokers have decreased BMD with increased risk fracture compared to nonsmokers.^13^, ^14^, ^15^

Smoking‐induced osteoporosis belongs to secondary osteoporosis, which refers to a systemic bone disease caused by long‐term smoking, such as reduced bone mass, degeneration of bone microstructure, and increased bone fragility. The pathogenesis of smoking induced osteoporosis mainly has the following aspects. First, nicotine in tobacco directly or indirectly stimulates the activity of osteoclasts and increases the concentration of blood calcium and urine calcium, leading to osteoporosis.^16^ Nicotine also induces apoptosis in human osteoblasts via a mechanism driven by H2O2 and entailing Glyoxalase 1‐dependent MG‐H1 accumulation leading to TG2‐mediated NF‐kB desensitization.^17^ Meanwhile, nicotine reduces estrogen synthesis, promotes estrogen dissociation and metabolism, and makes calcium regulated hormone dysregulation, thus affecting BMD.^18^ Besides, smoking destroys the stability of the bone marrow environment maintained by lymphocyte, leading to the reduction of bone marrow lymphocytes and changes in the immune system. The changes in bone marrow environment can induce the occurrence of osteoporosis.

#### 3.1.2 Reduced physical activity

Exercise plays an important role in regulating bone growth and development as well as bone metabolism.^19^, ^20^ Patients with COPD often stay in indoors due to dyspnea, respiratory failure and shortness of breath after activity in the later stage. Significantly reduced exercise ability is the most important cause of bone loss. A review by Lau et al.^21^ has indicated that “disuse” osteoporosis is the result of failure to achieve the optimal peak bone mass and strength. If disuse occurs during the period of bone mass accumulation, it leads to increased bone resorption and reduced bone formation. In ‘disuse’ osteoporosis, mechanical unloading is assumed to influence bone remodeling via change in insulin growth factor, bone morphogenetic proteins, parathyroid hormone and sclerostin.^22^, ^23^ It is reported that cycling power of 50% × 45 min increases plasma testosterone and free testosterone, which promotes the increase of the total amount of protein synthesis bone substrate, bone deposition, and bone thickening. Studies have shown that exercise also improves nerve and muscle function, muscle strength, weight gain, or maintenance, which is beneficial to promote bone replacement, prevent bone loss, and improve bone density and bone strength.^24^

#### 3.1.3 Low weight and sarcopenia

Body mass index (BMI) is an important physiological index used to judge the nutritional status of people and is closely related to BMD. Many studies have corroborated that low BMI and the presence of sarcopenia are associated with osteoporosis and fractures in COPD.^25^, ^26^, ^27^, ^28^ Low BMI and muscle wasting frequently present in severe COPD.^27^

Most patients with COPD have low body weight, which may be related to hypoxia, gastrointestinal congestion, reduced appetite and poor digestion and absorption function. A study^29^ including 104 patients with COPD has found that BMI less than or equal to 22 kg/m^2^ is associated with the incidence of osteoporosis, which indicates that poor nutritional status of COPD patients is more prone to osteoporosis. A low BMI changes the level of hormone that is responsible for maintaining bone cell metabolism and alter the bone turnover rate.^30^ Both fat and muscle mass provide mechanical loading on the weight‐bearing bones and facilitate bone formation.^31^

The decrease in BMD caused by malnutrition may be due to systemic inflammatory responses in COPD patients, such as TNF‐α, a proinflammatory cytokine, which causes malnutrition in COPD. TNF‐α is also an effective inhibitor of collagen synthesis and osteoclast bone resorption stimulator. On the other hand, adipokines secreted by adipocytes, such as leptin and adiponectin, or from the pancreatic β cells increase the proliferation and differentiation of osteoblasts, promote bone formation and regulate osteoclast development.^32^ It is also reported that the subjects of COPD with lower BMD show higher serum levels of RANK ligand and a higher ratio of RANK ligand/osteoprotegerin compared with those with normal BMD.^33^

In addition to low BMI, COPD is related to low fat‐free mass, reduced muscle strength and sarcopenia.^34^, ^35^, ^36^, ^37^ Several studies have shown that low fat‐free mass and sarcopenia in COPD patients are related with osteoporosis and increased fall risk, resulting in increased risk of fracture.^38^

### 3.2 Disease‐associated risk factors

#### 3.2.1 Systemic inflammatory

Systemic inflammatory response is considered to be the key to the co‐occurrence of COPD and osteoporosis.^39^, ^40^ Systemic inflammation in COPD may be the direct consequence of a systemic “spill‐over” of the ongoing pulmonary inflammation.^41^ Chronic airway inflammation is the characteristic of COPD. Neutrophils, macrophages, T lymphocytes, and other inflammatory cells are involved in the pathogenesis of COPD. Many cytokines induced by inflammatory cells are closely related to the occurrence of osteoporosis. They mainly include IL‐6,^42^, ^43^, ^44^ IL‐17,^45^ TNF‐α,^39^ OPG,^35^ and MMP.^46^, ^47^ These cytokines are well‐known inducers of osteoclasts both in vitro and in vivo and are considered to be involved in the pathogenesis of both primary and secondary osteoporosis^39^, ^48^ by regulating the RANKL (RANK/RANK ligand) ‐OPG axis system,^49^, ^50^ which leads to osteoporosis. RANK, RANKL, and OPG are members of the tumor necrosis factor receptor superfamily. Various cytokines and calcium‐related pathways are involved in bone remodeling and mineral metabolism. Moreover, systemic inflammation, represented by elevated CRP, is linked to osteoporosis in the general population.^51^, ^52^

#### 3.2.2 Glucocorticoid

Osteoporosis caused by long‐term use of glucocorticoid is the most common secondary osteoporosis. Its incidence is second only to postmenopausal osteoporosis and senile osteoporosis.^53^, ^54^ Glucocorticoid is currently an effective treatment for COPD, but it is associated with a reduction in BMD and an increased risk of fracture.^55^, ^56^

It is found^57^ that the fastest rate of bone loss occurred at 3–6 months after glucocorticoid treatment and bone loss increase with the increase of cumulative dose. Inhaled corticosteroid (ICS) is widely used for the regular treatment of COPD. However, studies investigated the effects of ICS on bone in patients of COPD show conflicting results. The difference is caused by dose and follow‐up time. Administered during acute exacerbations as GOLD guideline recommendations are relatively devoid of these adverse effects and ICS is not show to aggravate the bone mineral loss in COPD patients.^58^ However, according to a recent meta‐analysis including 16 RCTs with 17 513 subjects and seven observational studies with 69 000 subjects, ICS has been found to be associated with significant fracture risk (OR = 1.27 for RCTs and 1.21 for observational studies).^59^ Some other studies also have shown that ICS increases the risk of osteoporosis^34^, ^60^ and the loss of BMD are dose‐dependent and time‐dependent. Overall effects of ICS depend on the balance between its anti‐inflammatory effects and fracture risk.^61^ But studies have shown that the use of oral corticosteroids increase the risk of fracture.^55^, ^56^

Bone tissue is in the process of remodeling throughout life. The normal maintenance of this process depends on the balance between the osteogenic function of osteoblasts and the bone resorption function of osteoclasts.^62^, ^63^ Glucocorticoid can directly acts on bone tissue, promotes osteoblast apoptosis through WNT signaling and inhibits osteoblast precursor differentiation and osteoblast maturation through IGF‐1, MIF, and other cytokines.^64^ On the other hand, glucocorticoid also increases the number and the activity of osteoclasts by affecting cytokines such as RANKL/RANK/OPG, PTH, and GP130,^65^ thus increasing bone resorption. It also regulates the metabolism of vitamin D by affecting 1,25 (OH) 2D3 and reduces the absorption of intestinal calcium.^66^ Glucocorticoid increases serum parathyroid hormone level, reduces calcium transport function of intestinal mucosa, reduces calcium absorption, and inhibits renal calcium ion reabsorption.^67^ Inhibition of pituitary secretion of adrenocorticotrophic hormone decreases sex hormone levels, in which the reduction of estrogen level promotes osteoclast formation and increases bone resorption. In conclusion, glucocorticoid affects bone metabolism in a variety of ways, leading to bone loss and inducing osteoporosis.

#### 3.2.3 Vitamin D deficiency

Vitamin D is an essential part of human hormone. It stabilizes concentration of serum calcium phosphate. Low blood calcium concentration induces parathyroid hormone secretion, which is released to the kidney and affects the absorption and storage of calcium and phosphorus. According to the Endocrine Society Clinical Practice Guideline, vitamin D deficiency and insufficiency are defined as 25‐hydroxy Vitamin D levels below 20 ng/ml and 20–30 ng/ml, respectively.^68^ Vitamin D deficiency is consistently reported to be more common in patients with COPD than in healthy controls.^69^, ^70^, ^71^ Vitamin D deficiency in patients with COPD may be related to the following factors: poor dietary habits, reduced synthetic ability due to skin aging, decreased sun exposure due to restricted activities, renal dysfunction, and increased vitamin D metabolism due to use glucocorticoid. In summary, the intake, synthesis, storage, and metabolism of vitamin D are all disrupted. The body cannot maintain calcium homeostasis in the low 25(OH)D3 state. The mineralized collagen matrix in bone further decomposes and the beneficial functions of anti‐oxidation and anti‐infection also lost, which leads to the decrease of bone mass.

#### 3.2.4 Hypoxemia and hypercapnia

The patients with COPD have a large number of loss of alveoli and capillaries, reduced diffusion area, ventilation and blood flow ratio imbalance. Ventilation dysfunction leads to hypoxia and carbon dioxide retention and further causes varying degrees of hypoxemia and hypercapnia. Studies have proved that oxygen concentration has a significant effect on the formation of osteoclasts in mouse bone marrow cells. With the reduction of oxygen concentration, the differentiation of preosteoclasts into osteoclasts significantly increases.^72^ Steinbrech et al.^72^ have believed that the effect of hypoxia on osteoblasts is mainly through the effect of vascular endothelial growth factor on angiogenesis under the control of hypoxia‐inducible factor 1α(HIF‐1α). Angiogenesis and bone formation interact and influence each other. HIF‐1α also affects osteoblast formation through bone morphogenetic protein, prostaglandin 2, and its receptor EP1.^73^

The oxidative process of cell metabolism is impaired and ATP synthesis in mitochondria is insufficient in the state of hypoxemia.^74^ Collagen synthase function is affected and collagen synthesis and osteoblast activity in vivo are affected. In addition, Knowles et al.^75^ have shown that hypoxia stimulate the differentiation of monocyte progenitor cells into osteoclasts, which can stimulates the formation of osteoclasts.

#### 3.2.5 Anemia

Anemia is commonly seen in patients with COPD and is related with a low‐grade systemic inflammation. Elevated osteoclast activity and subsequent accelerated bone resorption are proposed as underlying mechanisms of osteoporosis in anemia patients. The reduced blood volume stimulates the proliferation of hematopoietic cells, including osteoclasts. Proliferated osteoclasts stimulate bone resorption. Although osteoblast formation is also stimulated by blood loss, stimulated bone resorption may hinders bone remodeling cycles and results osteoblast fatigue.^76^

Hypoxemia in anemic patients mediates the risk of osteoporosis. Chronic hypoxia increases oxidative stress, and acidification of the extracellular matrix impairs bone metabolism.^77^, ^78^ Although anemia appears to be linked with bone health, the effects of anemia on bone remodeling are not entirely clear yet. Additional research is necessary to be elucidated whether anemia is an independent factor.

#### 3.2.6 Therapy

The clinical treatment of COPD patients mainly focused on the lung function and oxygenation capacity of patients, but ignores the prevention and treatment of osteoporosis. However, osteoporosis has the same serious consequences as COPD, clinical attention should be paid to it. The treatment of osteoporosis emphasizes comprehensive intervention, which mainly include basic treatment and anti‐osteoporosis drugs.

#### 3.2.7 Change lifestyle

On the one hand, excessive use or abuse of alcohol,^15^, ^79^, ^80^, ^81^, ^82^ caffeine,^83^, ^84^ and carbonated drinks^83^ should be avoided, which are harmful for both health people and patients with osteoporosis. On the other hand, any form of nicotine should be discouraged.^85^, ^86^ Besides, patients with COPD and osteoporosis should be given a balanced diet. It is recommended that patients eat foods with high calcium and foods rich in vitamin D, such as egg yolk and liver.^83^ Protein and vitamin C increase the absorption of calcium in the body, so patients are recommended to eat lean meat, fish, beans, milk and vitamin‐rich vegetables and fruits.^83^, ^87^, ^88^ At the same time, sufficient sunshine should be kept to promote the absorption of calcium.^80^, ^89^ It is recommended that we can be exposed to the sun for 15 to 30 min from 11:00 AM to 15:00 PM.

#### 3.2.8 Pulmonary/exercise rehabilitation

A sedentary lifestyle and prolonged rest in bed lead to bone loss in the involutional period. Therefore, we encourage physical activity and implement a moderate exercise program to minimize bone loss in elderly people.^90^, ^91^ Exercise shows promise as a non‐invasive and non‐pharmacological method of regulating both osteoporosis. Physical exercise effectively decreases risk factors for falling and improve balance.^92^, ^93^ In the past years, many studies^94^, ^95^ have reported consistent results on the beneficial effects of exercise on BMD. Mechanical signals produced by exercise can promote bone and muscle anabolism.^96^ In general, therapeutic exercises for osteoporosis can be ranked in two types of activities. One is weight‐bearing aerobic exercises, such as walking, stair climbing, jogging, volleyball, tennis, tai chi, and dancing. Walking predominating as the most common form of physical activity in older adults,^97^ while daily walking activity is associated with a range of positive health outcomes, its potential for increasing or maintaining BMD is less convincing.^95^ Another is strength or resistance training,^98^ in which the joints are moved against some kind of resistance, in the form of free weights, machines or one's own body weight and develop muscle hypertrophy and strength.^99^, ^100^, ^101^, ^102^ Huovinen et al. has demonstrated that a 16‐week resistance training intervention, involving exercises such as abdominal crunches, leg presses and other large muscle group exercises improves total hip BMD by 6%.^103^

The international consensus is that rehabilitation programs are an important part of COPD treatment,^3^ which follows from the realization that drug therapy for COPD is inadequate. A vicious cycle of deterioration in physical capacity, shortness of breath, anxiety is formed in patient with COPD. Rehabilitation is beneficial in improving health‐related quality of life and exercise capacity breaks this cycle by introducing physical training, psychological support and networking with other COPD patients.^35^, ^104^ All patients with COPD can benefit from physical training.^105^, ^106^

A study including 65 RCTs and involving 3822 participants has found statistically significant improvement for all included outcomes. In four important domains of quality of life (Chronic Respiratory Questionnaire scores for dyspnea, fatigue, emotional function, and mastery).^104^ In particular, physical exercise^105^, ^106^ has been shown to improve general conditions of COPD patients and to significantly increase BMD. Evidence has shown that aerobic exercise increase BMD, while a combination of resistance training and balance training prevent the risk of falls and fractures in elderly people.^92^, ^93^

Lacking of time and access to transportation are the most commonly reported barriers to exercise participation in patients with osteoporosis. Thus, clinicians and researchers should explore strategy to facilitate exercise participation in this population, such as the safety and efficacy of home‐based impact exercise.

#### 3.2.9 Drug therapy

As for pharmacological intervention, adequate amounts of vitamin D and calcium are first recommended.^107^ Osteoporosis guidelines recommend that daily intake is 1000–1200 mg for calcium and 800–1000 units for vitamin D3.^108^ However, hydroxylated vitamin D metabolites increase the risk of hypercalcemia and hypercalciuria, they therefore need to monitor with serial serum and urinary calcium measurement.^109^ There have many drugs for treatment of primary osteoporosis. However, there are few studies on pharmacological intervention for COPD‐associated osteoporosis.^110^, ^111^ Because of lacking specific evidence in COPD patients, it is recommended to basically follow general practice guidelines for the treatment of primary osteoporosis^6^, ^112^, ^113^ First‐line treatment includes bisphosphonates such as alendronate, risedronate and zoledronate, denosumab, and teriparatide. We use mathematical algorithms that quantify the risk in terms of “10‐year fracture risk” such as FRAX. Oral bisphosphonates can be considered if the patient has low to moderate risk of fracture. If patient has high risk of fracture or has osteoporotic fracture, intravenous bisphosphonates are largely recommended.^109^, ^114^

## 4 CONCLUSIONS

Osteoporosis is very common in patients with COPD and has profound impact on the quality of life in COPD patients, but COPD associated osteoporosis is extremely underdiagnosed and undertreated. Thus, we propose to act immediately to screen every COPD subject for osteoporosis, identify patients at high risk of fracture and treat them with the standard medications.

**List of Abbreviations**
BMD: bone mineral density
BMI: body mass index
COPD: chronic obstructive pulmonary disease
EP1: prostaglandin E1
GP130: glycoprotein130
HIF‐1α: hypoxia‐inducible factor 1α
ICS: inhaled corticosteroid
IGF‐1: insulin‐like growth factor‐1
IL: interleukin
MIF: migration inhibition factor
MMP: matrix metalloproteinase
OPG: osteoprotegerin
OR: odds ratio
PTH: parathyroid hormone
RANK: receptor activator of nuclear factor kappa
RCT: randomized controlled trial
TNF‐α: tumor necrosis factor α

## CONFLICT OF INTEREST

The author declare that they have no competing interests.

## ETHICS STATEMENT

Ethics statement is not applicable.

## AUTHOR CONTRIBUTIONS

YJL, HCG, and JRW contributed substantially to the article concept. YJL and HCG retrieved literature and manuscript writing. LZ and JRW revised the manuscript. LZ and JRW reviewed and approved the final version before submission. All the listed authors have participated actively in the study. All authors read and approved the final manuscript.

## Data Availability

Data availability statement is not applicable.

## References

[1] Wang C , Xu J , Yang L , et al. Prevalence and risk factors of chronic obstructive pulmonary disease in China (the China Pulmonary Health [CPH] study): a national cross‐sectional study. Lancet (London, England). 2018;391(10131):1706‐1717. doi:10.1016/S0140-6736(18)30841-9 29650248

[2] Celli BR , Agustí A . COPD: time to improve its taxonomy? ERJ Open Res. 2018;4(1):00132‐02017. doi:10.1183/23120541.00132-2017 PMC5912933 29707563

[3] Singh D , Agusti A , Anzueto A , et al. Global strategy for the diagnosis, management, and prevention of chronic obstructive lung disease: the GOLD science committee report 2019. Eur Respir J. 2019;53(5):1900164. doi:10.1183/13993003.00164-2019 30846476

[4] Iheanacho I , Zhang S , King D , Rizzo M , Ismaila AS . Economic burden of chronic obstructive pulmonary disease (COPD): a systematic literature review. Int J Chron Obstruct Pulmon Dis. 2020;15:439‐460. doi:10.2147/COPD.S234942 32161455 PMC7049777

[5] Lehouck A , Boonen S , Decramer M , Janssens W . COPD, bone metabolism, and osteoporosis. Chest. 2011;139(3):648‐657. doi:10.1378/chest.10-1427 21362651

[6] Osteoporosis prevention, diagnosis, and therapy. JAMA. 2001;285(6):785‐795. doi:10.1001/jama.285.6.785 11176917

[7] Kanis JA , McCloskey EV , Johansson H , Oden A , Melton LJ 3rd , Khaltaev N . A reference standard for the description of osteoporosis. Bone. 2008;42(3):467‐475. doi:10.1016/j.bone.2007.11.001 18180210

[8] Sözen T , Özışık L , Başaran N . An overview and management of osteoporosis. Eur J Rheumatol. 2017;4(1):46‐56. doi:10.5152/eurjrheum.2016.048 28293453 PMC5335887

[9] Chen Y , Ramsook A , Coxson H , Bon J , Reid W . Prevalence and risk factors for osteoporosis in individuals with COPD: a systematic review and meta‐analysis. Chest. 2019;156(6):1092‐1110. doi:10.1016/j.chest.2019.06.036 31352034

[10] Schnell K , Weiss CO , Lee T , et al. The prevalence of clinically‐relevant comorbid conditions in patients with physician‐diagnosed COPD: a cross‐sectional study using data from NHANES 1999‐2008. BMC Pulm Med. 2012;12(1):26. doi:10.1186/1471-2466-12-26 22695054 PMC3461433

[11] Jaramillo JD , Wilson C , Stinson DS , et al. Reduced bone density and vertebral fractures in smokers. Men and COPD patients at increased risk. Ann Am Thorac Soc. 2015;12(5):648‐656. doi:10.1513/AnnalsATS.201412-591OC 25719895 PMC4418341

[12] Bitar AN , Syed Sulaiman SA , Ali IAH , Khan I , Khan AH . Osteoporosis among patients with chronic obstructive pulmonary disease: systematic review and meta‐analysis of prevalence, severity, and therapeutic outcomes. J Pharm Bioallied Sci. 2019;11(4):310‐320. doi:10.4103/jpbs.JPBS_126_19 31619912 PMC6791086

[13] Pompe E , Bartstra J , Verhaar HJ , et al. Bone density loss on computed tomography at 3‐year follow‐up in current compared to former male smokers. Eur J Radiol. 2017;89:177‐181. doi:10.1016/j.ejrad.2017.02.011 28267536

[14] Bijelic R , Milicevic S , Balaban J . Risk factors for osteoporosis in postmenopausal women. Med Arch (Sarajevo, Bosnia and Herzegovina). 2017;71(1):25‐28. doi:10.5455/medarh.2017.71.25-28 PMC5364787 28428669

[15] Yang CY , Cheng‐Yen Lai J , Huang WL , Hsu CL , Chen SJ . Effects of sex, tobacco smoking, and alcohol consumption osteoporosis development: evidence from Taiwan biobank participants. Tob Induc Dis. 2021;19(June):52‐58. doi:10.18332/tid/136419 34177414 PMC8210532

[16] Law M , Hackshaw A . A meta‐analysis of cigarette smoking, bone mineral density and risk of hip fracture: recognition of a major effect. BMJ (Clin Res Ed). 1997;315(7112):841‐846. doi:10.1136/bmj.315.7112.841 PMC2127590 9353503

[17] Marinucci L , Balloni S , Fettucciari K , Bodo M , Talesa VN , Antognelli C . Nicotine induces apoptosis in human osteoblasts via a novel mechanism driven by H(2)O(2) and entailing glyoxalase 1‐dependent MG‐H1 accumulation leading to TG2‐mediated NF‐kB desensitization: implication for smokers‐related osteoporosis. Free Radic Biol Med. 2018;117:6‐17. doi:10.1016/j.freeradbiomed.2018.01.017 29355739

[18] Papakitsou E , Margioris A , Dretakis K , et al. Body mass index (BMI) and parameters of bone formation and resorption in postmenopausal women. Maturitas. 2004;47(3):185‐193. doi:10.1016/S0378-5122(03)00282-2 15036488

[19] Santos L , Elliott‐Sale KJ , Sale C . Exercise and bone health across the lifespan. Biogerontology. 2017;18(6):931‐946. doi:10.1007/s10522-017-9732-6 29052784 PMC5684300

[20] Yuan Y , Chen X , Zhang L , et al. The roles of exercise in bone remodeling and in prevention and treatment of osteoporosis. Prog Biophys Mol Biol. 2016;122(2):122‐130. doi:10.1016/j.pbiomolbio.2015.11.005 26657214

[21] Lau RY , Guo X . A review on current osteoporosis research: with special focus on disuse bone loss. J Osteoporos. 2011;2011:293808. doi:10.4061/2011/293808 21876833 PMC3160709

[22] Canalis E . Management of endocrine disease: novel anabolic treatments for osteoporosis. Eur J Endocrinol. 2018;178(2):R33‐r44. doi:10.1530/EJE-17-0920 29113980 PMC5819362

[23] Hinton PS , Nigh P , Thyfault J . Serum sclerostin decreases following 12 months of resistance‐ or jump‐training in men with low bone mass. Bone. 2017;96:85‐90. doi:10.1016/j.bone.2016.10.011 27744012 PMC5328803

[24] Mena‐Montes B , Hernández‐Álvarez D , Pedraza‐Vázquez G , et al. Low‐intensity exercise routine for a long period of time prevents osteosarcopenic obesity in sedentary old female rats, by decreasing inflammation and oxidative stress and increasing GDF‐11. Oxid Med Cell Longev. 2021;2021:5526665. doi:10.1155/2021/5526665 34336096 PMC8315843

[25] Lee SH , Kwon HY . Prevalence of osteoporosis in Korean patients with chronic obstructive pulmonary disease and their health‐related quality of life according to the Korea National Health and Nutrition Examination Survey 2008‐2011. J Bone Metab. 2017;24(4):241‐248. doi:10.11005/jbm.2017.24.4.241 29259964 PMC5734950

[26] Kim SW , Lee JM , Ha JH , et al. Association between vitamin D receptor polymorphisms and osteoporosis in patients with COPD. Int J Chron Obstruct Pulmon Dis. 2015;10:1809‐1817. doi:10.2147/COPD.S91576 26379431 PMC4567171

[27] Lin CW , Chen YY , Chen YJ , Liang CY , Lin MS , Chen W . Prevalence, risk factors, and health‐related quality of life of osteoporosis in patients with COPD at a community hospital in Taiwan. Int J Chron Obstruct Pulmon Dis. 2015;10:1493‐1500. doi:10.2147/COPD.S85432 26251589 PMC4524376

[28] Espallargues M , Sampietro‐Colom L , Estrada MD , et al. Identifying bone‐mass‐related risk factors for fracture to guide bone densitometry measurements: a systematic review of the literature. Osteoporos Int. 2001;12(10):811‐822. doi:10.1007/s001980170031 11716183

[29] McEvoy C , Ensrud K , Bender E , et al. Association between corticosteroid use and vertebral fractures in older men with chronic obstructive pulmonary disease. Am J Respir Crit Care Med. 1998;157(3):704‐709. doi:10.1164/ajrccm.157.3.9703080 9517579

[30] Rosen CJ , Klibanski A . Bone, fat, and body composition: evolving concepts in the pathogenesis of osteoporosis. Am J Med. 2009;122(5):409‐414. doi:10.1016/j.amjmed.2008.11.027 19375545

[31] Hamrick MW , McNeil PL , Patterson SL . Role of muscle‐derived growth factors in bone formation. J Musculoskelet Neuronal Interact. 2010;10(1):64‐70. 20190381 PMC3753580

[32] Reid IR . Fat and bone. Arch Biochem Biophys. 2010;503(1):20‐27. doi:10.1016/j.abb.2010.06.027 20599663

[33] Braun T , Schett G . Pathways for bone loss in inflammatory disease. Curr Osteoporos Rep. 2012;10(2):101‐108. doi:10.1007/s11914-012-0104-5 22527726

[34] Duckers JM , Evans BA , Fraser WD , Stone MD , Bolton CE , Shale DJ . Low bone mineral density in men with chronic obstructive pulmonary disease. Respir Res. 2011;12(1):101. doi:10.1186/1465-9921-12-101 21812978 PMC3161864

[35] Ugay L , Kochetkova E , Nevzorova V , Maistrovskaia Y . Role of osteoprotegerin and receptor activator of nuclear factor‐κB ligand in bone loss related to advanced chronic obstructive pulmonary disease. Chin Med J (Engl). 2016;129(14):1696‐1703. doi:10.4103/0366-6999.185857 27411457 PMC4960959

[36] Hwang JA , Kim YS , Leem AY , et al. Clinical implications of sarcopenia on decreased bone density in men with COPD. Chest. 2017;151(5):1018‐1027. doi:10.1016/j.chest.2016.12.006 28012805

[37] Lee DW , Jin HJ , Shin KC , Chung JH , Lee HW , Lee KH . Presence of sarcopenia in asthma‐COPD overlap syndrome may be a risk factor for decreased bone‐mineral density, unlike asthma: Korean National Health and Nutrition Examination Survey (KNHANES) IV and V (2008–2011). Int J Chron Obstruct Pulmon Dis. 2017;12:2355‐2362. doi:10.2147/COPD.S138497 28848336 PMC5557102

[38] Jang SY , Park J , Ryu SY , Choi SW . Low muscle mass is associated with osteoporosis: a nationwide population‐based study. Maturitas. 2020;133:54‐59. doi:10.1016/j.maturitas.2020.01.003 32005424

[39] Liang B , Feng Y . The association of low bone mineral density with systemic inflammation in clinically stable COPD. Endocrine. 2012;42(1):190‐195. doi:10.1007/s12020-011-9583-x 22198912

[40] Lin CH , Chen KH , Chen CM , Chang CH , Huang TJ , Lin CH . Risk factors for osteoporosis in male patients with chronic obstructive pulmonary disease in Taiwan. PeerJ. 2018;6:e4232 doi:10.7717/peerj.4232 29340241 PMC5768161

[41] Fabbri LM , Rabe KF . From COPD to chronic systemic inflammatory syndrome? Lancet (London, England). 2007;370(9589):797‐799. doi:10.1016/S0140-6736(07)61383-X 17765529

[42] Raisz LG . Pathogenesis of osteoporosis: concepts, conflicts, and prospects. J Clin Invest. 2005;115(12):3318‐3325. doi:10.1172/JCI27071 16322775 PMC1297264

[43] Xiong Z , Leme AS , Ray P , Shapiro SD , Lee JS . CX3CR1+ lung mononuclear phagocytes spatially confined to the interstitium produce TNF‐α and IL‐6 and promote cigarette smoke‐induced emphysema. J Immunol (Baltimore, Md: 1950). 2011;186(5):3206‐3214. doi:10.4049/jimmunol.1003221 PMC3912553 21278339

[44] Ruwanpura SM , McLeod L , Miller A , et al. Interleukin‐6 promotes pulmonary emphysema associated with apoptosis in mice. Am J Respir Cell Mol Biol. 2011;45(4):720‐730. doi:10.1165/rcmb.2010-0462OC 21297079

[45] Chen K , Pociask DA , McAleer JP , et al. IL‐17RA is required for CCL2 expression, macrophage recruitment, and emphysema in response to cigarette smoke. PLoS ONE. 2011;6(5):e20333. doi:10.1371/journal.pone.0020333 21647421 PMC3103542

[46] Vitenberga Z , Pilmane M , Babjoniševa A . The evaluation of inflammatory, anti‐inflammatory and regulatory factors contributing to the pathogenesis of COPD in airways. Pathol Res Pract. 2019;215(1):97‐105. doi:10.1016/j.prp.2018.10.029 30392917

[47] Bolton CE , Stone MD , Edwards PH , Duckers JM , Evans WD , Shale DJ . Circulating matrix metalloproteinase‐9 and osteoporosis in patients with chronic obstructive pulmonary disease. Chron Respir Dis. 2009;6(2):81‐87. doi:10.1177/1479972309103131 19411568

[48] Bai P , Sun Y , Jin J , et al. Disturbance of the OPG/RANK/RANKL pathway and systemic inflammation in COPD patients with emphysema and osteoporosis. Respir Res. 2011;12(1):157. doi:10.1186/1465-9921-12-157 22176920 PMC3260206

[49] Afonso AS , Verhamme KM , Sturkenboom MC , Brusselle GG . COPD in the general population: prevalence, incidence and survival. Respir Med. 2011;105(12):1872‐1884. doi:10.1016/j.rmed.2011.06.012 21852081

[50] Hardy R , Cooper MS . Bone loss in inflammatory disorders. J Endocrinol. 2009;201(3):309‐320. doi:10.1677/JOE-08-0568 19443863

[51] Hoepers AT , Menezes MM , Fröde TS . Systematic review of anaemia and inflammatory markers in chronic obstructive pulmonary disease. Clin Exp Pharmacol Physiol. 2015;42(3):231‐239. doi:10.1111/1440-1681.12357 25641228

[52] Bade G , Khan MA , Srivastava AK , et al. Serum cytokine profiling and enrichment analysis reveal the involvement of immunological and inflammatory pathways in stable patients with chronic obstructive pulmonary disease. Int J Chron Obstruct Pulmon Dis. 2014;9:759‐773. doi:10.2147/COPD.S61347 25125975 PMC4130715

[53] Compston J . Glucocorticoid‐induced osteoporosis: an update. Endocrine. 2018;61(1):7‐16. doi:10.1007/s12020-018-1588-2 29691807 PMC5997116

[54] Mazziotti G , Angeli A , Bilezikian JP , Canalis E , Giustina A . Glucocorticoid‐induced osteoporosis: an update. Trends Endocrinol Metab. 2006;17(4):144‐149. doi:10.1016/j.tem.2006.03.009 16678739

[55] Amiche MA , Albaum JM , Tadrous M , et al. Fracture risk in oral glucocorticoid users: a Bayesian meta‐regression leveraging control arms of osteoporosis clinical trials. Osteoporos Int. 2016;27(5):1709‐1718. doi:10.1007/s00198-015-3455-9 26694595

[56] Scanlon PD , Connett JE , Wise RA , et al. Loss of bone density with inhaled triamcinolone in Lung Health Study II. Am J Respir Crit Care Med. 2004;170(12):1302‐1309. doi:10.1164/rccm.200310-1349OC 15374846

[57] Suzuki Y , Sato S . Secondary osteoporosis update. Clinical significance of glucocorticoid‐induced osteoporosis. Clin Calcium. 2010;20(5):645‐653. PMID: 20445275. 20445275

[58] Wüst RC , Degens H . Factors contributing to muscle wasting and dysfunction in COPD patients. Int J Chron Obstruct Pulmon Dis. 2007;2(3):289‐300. 18229567 PMC2695204

[59] Loke YK , Cavallazzi R , Singh S . Risk of fractures with inhaled corticosteroids in COPD: systematic review and meta‐analysis of randomised controlled trials and observational studies. Thorax. 2011;66(8):699‐708. doi:10.1136/thx.2011.160028 21602540

[60] Chiu KL , Lee CC , Chen CY . Evaluating the association of osteoporosis with inhaled corticosteroid use in chronic obstructive pulmonary disease in Taiwan. Sci Rep. 2021;11(1):724. doi:10.1038/s41598-020-80815-y 33436973 PMC7804267

[61] Mathioudakis AG , Amanetopoulou SG , Gialmanidis IP , et al. Impact of long‐term treatment with low‐dose inhaled corticosteroids on the bone mineral density of chronic obstructive pulmonary disease patients: aggravating or beneficial? Respirology. 2013;18(1):147‐153. doi:10.1111/j.1440-1843.2012.02265.x 22985270

[62] Harada S , Rodan G . Control of osteoblast function and regulation of bone mass. Nature. 2003;423(6937):349‐355. doi:10.1038/nature01660 12748654

[63] Boyle W , Simonet W , Lacey D . Osteoclast differentiation and activation. Nature. 2003;423(6937):337‐342. doi:10.1038/nature01658 12748652

[64] Seckl JR . 11beta‐hydroxysteroid dehydrogenases: changing glucocorticoid action. Curr Opin Pharmacol. 2004;4(6):597‐602. doi:10.1016/j.coph.2004.09.001 15525550

[65] Lu P , Yang Y , Guo S , Yang T . Factors associated with osteoporosis in patients with chronic obstructive pulmonary disease—a nationwide retrospective study. Osteoporos Int. 2017;28(1):359‐367. doi:10.1007/s00198-016-3732-2 27519532

[66] Kjensli A , Mowinckel P , Ryg M , Falch J . Low bone mineral density is related to severity of chronic obstructive pulmonary disease. Bone. 2007;40(2):493‐497. doi:10.1016/j.bone.2006.09.005 17049326

[67] Mazziotti G , Formenti AM , Adler RA , et al. Glucocorticoid‐induced osteoporosis: pathophysiological role of GH/IGF‐I and PTH/VITAMIN D axes, treatment options and guidelines. Endocrine. 2016;54(3):603‐611. doi:10.1007/s12020-016-1146-8 27766553

[68] Holick MF , Binkley NC , Bischoff‐Ferrari HA , et al. Evaluation, treatment, and prevention of vitamin D deficiency: an Endocrine Society clinical practice guideline. J Clin Endocrinol Metab. 2011;96(7):1911‐1930. doi:10.1210/jc.2011-0385 21646368

[69] Janssens W , Bouillon R , Claes B , et al. Vitamin D deficiency is highly prevalent in COPD and correlates with variants in the vitamin D‐binding gene. Thorax. 2010;65(3):215‐220. doi:10.1136/thx.2009.120659 19996341

[70] Kokturk N , Baha A , Oh YM , Young Ju J , Jones PW . Vitamin D deficiency: what does it mean for chronic obstructive pulmonary disease (COPD)? A comprehensive review for pulmonologists. Clin Respir J. 2018;12(2):382‐397. doi:10.1111/crj.12588 27925404

[71] Graat‐Verboom L , Smeenk FW , van den Borne BE , et al. Progression of osteoporosis in patients with COPD: a 3‐year follow up study. Respir Med. 2012;106(6):861‐870. doi:10.1016/j.rmed.2011.12.020 22369986

[72] Gorissen B , de Bruin A , Miranda‐Bedate A , et al. Hypoxia negatively affects senescence in osteoclasts and delays osteoclastogenesis. J Cell Physiol. 2018;234(1):414‐426. doi:10.1002/jcp.26511 29932209 PMC6220985

[73] Chen D , Li Y , Zhou Z , et al. HIF‐1α inhibits Wnt signaling pathway by activating Sost expression in osteoblasts. PLoS ONE. 2013;8(6):e65940. doi:10.1371/journal.pone.0065940 23776575 PMC3679053

[74] Jorgensen C , Khoury M . Musculoskeletal progenitor/stromal cell‐derived mitochondria modulate cell differentiation and therapeutical function. Front Immunol. 2021;12:606781. doi:10.3389/fimmu.2021.606781 33763061 PMC7982675

[75] Knowles H , Athanasou N . Hypoxia‐inducible factor is expressed in giant cell tumour of bone and mediates paracrine effects of hypoxia on monocyte‐osteoclast differentiation via induction of VEGF. J Pathol. 2008;215(1):56‐66. doi:10.1002/path.2319 18283716

[76] Gurevitch O , Slavin S . The hematological etiology of osteoporosis. Med Hypotheses. 2006;67(4):729‐735. doi:10.1016/j.mehy.2006.03.051 16797864

[77] Fujimoto H , Fujimoto K , Ueda A , Ohata M . Hypoxemia is a risk factor for bone mass loss. J Bone Miner Metab. 1999;17(3):211‐216. doi:10.1007/s007740050087 10757682

[78] Karadag F , Cildag O , Yurekli Y , Gurgey O . Should COPD patients be routinely evaluated for bone mineral density? J Bone Miner Metab. 2003;21(4):242‐246. 12811630 10.1007/s00774-002-0416-0

[79] Naruo M , Negishi Y , Okuda T , Katsuyama M , Okazaki K , Morita R . Alcohol consumption induces murine osteoporosis by downregulation of natural killer T‐like cell activity. Immun Inflamm Dis. 2021;9(4):1370‐1382. doi:10.1002/iid3.485 34214248 PMC8589379

[80] Min CY , Yoo DM , Choi HG . Associations between physical activity, sunshine duration and osteoporosis according to obesity and other lifestyle factors: a nested case‐control study. Int J Environ Res Public Health. 2021;18(9). doi:10.3390/ijerph18094437 PMC8122401 33922027

[81] Pasco JA , Anderson KB , Hyde NK , Williams LJ , Rufus‐Membere P , Holloway‐Kew KL . High alcohol intake in older men and the probability of osteoporotic fracture according to the FRAX algorithm. Nutrients. 2021;13(9). doi:10.3390/nu13092955 PMC8468672 34578830

[82] Luo Z , Liu Y , Liu Y , Chen H , Shi S , Liu Y . Cellular and molecular mechanisms of alcohol‐induced osteopenia. Cell Molec Life Sci. 2017;74(24):4443‐4453. doi:10.1007/s00018-017-2585-y 28674727 PMC11107754

[83] Feng W , Wang X , Huang D , Lu A . Role of diet in osteoporosis incidence: umbrella review of meta‐analyses of prospective observational studies. Crit Rev Food Sci Nutr. 2021;1‐10. doi:10.1080/10408398.2021.1989374 34644187

[84] Li S , Dai Z , Wu Q . Effect of coffee intake on hip fracture: a meta‐analysis of prospective cohort studies. Nutr J. 2015;14(1):38. doi:10.1186/s12937-015-0025-0 25928220 PMC4412140

[85] Thorin MH , Wihlborg A , Åkesson K , Gerdhem P . Smoking, smoking cessation, and fracture risk in elderly women followed for 10 years. Osteoporos Int. 2016;27(1):249‐255. doi:10.1007/s00198-015-3290-z 26302684

[86] Vestergaard P , Mosekilde L . Fracture risk associated with smoking: a meta‐analysis. J Intern Med. 2003;254(6):572‐583. doi:10.1111/j.1365-2796.2003.01232.x 14641798

[87] Dawson‐Hughes B . Acid‐base balance of the diet‐implications for bone and muscle. Eur J Clin Nutr. 2020;74(Suppl 1):7‐13. doi:10.1038/s41430-020-0691-7 32873951

[88] Movassagh EZ , Vatanparast H . Current evidence on the association of dietary patterns and bone health: a scoping review. Adv Nutr (Bethesda, Md). 2017;8(1):1‐16. doi:10.3945/an.116.013326 PMC5227978 28096123

[89] Lee HJ , Kim CO , Lee DC . Association between daily sunlight exposure and fractures in older Korean adults with osteoporosis: a nationwide population‐based cross‐sectional study. Yonsei Med J. 2021;62(7):593‐599. doi:10.3349/ymj.2021.62.7.593 34164956 PMC8236351

[90] Jain RK , Vokes T . Physical activity as measured by accelerometer in NHANES 2005‐2006 is associated with better bone density and trabecular bone score in older adults. Arch Osteoporos. 2019;14(1):29. doi:10.1007/s11657-019-0583-4 30826896

[91] Madani A , Alack K , Richter MJ , Krüger K . Immune‐regulating effects of exercise on cigarette smoke‐induced inflammation. J Inflamm Res. 2018;11:155‐167. doi:10.2147/JIR.S141149 29731655 PMC5923223

[92] Sadeghi H , Jehu DA , Daneshjoo A , et al. Effects of 8 weeks of balance training, virtual reality training, and combined exercise on lower limb muscle strength, balance, and functional mobility among older men: a randomized controlled trial. Sports Health. 2021;13(6):606‐612. doi:10.1177/1941738120986803 33583253 PMC8558995

[93] Gronlund C , Christoffersen KS , Thomsen K , Masud T , Jepsen DB , Ryg J . Effect of blood‐flow restriction exercise on falls and fall related risk factors in older adults 60 years or above: a systematic review. J Musculoskelet Neuronal Interact. 2020;20(4):513‐525. 33265079 PMC7716683

[94] Hingorjo MR , Zehra S , Saleem S , Qureshi MA . Serum Interleukin‐15 and its relationship with adiposity Indices before and after short‐term endurance exercise. Pakistan J Med Sci. 2018;34(5):1125‐1131. doi:10.12669/pjms.345.15516 PMC6191778 30344562

[95] Langsetmo L , Hitchcock CL , Kingwell EJ , et al. Physical activity, body mass index and bone mineral density‐associations in a prospective population‐based cohort of women and men: the Canadian Multicentre Osteoporosis Study (CaMos). Bone. 2012;50(1):401‐408. doi:10.1016/j.bone.2011.11.009 22154839 PMC3737114

[96] Pagnotti G , Styner M , Uzer G , et al. Combating osteoporosis and obesity with exercise: leveraging cell mechanosensitivity. Nat Rev Endocrinol. 2019;15(6):339‐355. doi:10.1038/s41574-019-0170-1 30814687 PMC6520125

[97] Hulteen RM , Smith JJ , Morgan PJ , et al. Global participation in sport and leisure‐time physical activities: a systematic review and meta‐analysis. Prev Med. 2017;95:14‐25. doi:10.1016/j.ypmed.2016.11.027 27939265

[98] Kitsuda Y , Wada T , Noma H , Osaki M , Hagino H . Impact of high‐load resistance training on bone mineral density in osteoporosis and osteopenia: a meta‐analysis. J Bone Miner Metab. 2021;39(5):787‐803. doi:10.1007/s00774-021-01218-1 33851269

[99] Watson SL , Weeks BK , Weis LJ , Horan SA , Beck BR . Heavy resistance training is safe and improves bone, function, and stature in postmenopausal women with low to very low bone mass: novel early findings from the LIFTMOR trial. Osteoporos Int. 2015;26(12):2889‐2894. doi:10.1007/s00198-015-3263-2 26243363

[100] Hinton PS , Nigh P , Thyfault J . Effectiveness of resistance training or jumping‐exercise to increase bone mineral density in men with low bone mass: a 12‐month randomized, clinical trial. Bone. 2015;79:203‐212. 26092649 10.1016/j.bone.2015.06.008 PMC4503233

[101] Zhao R , Zhao M , Xu Z . The effects of differing resistance training modes on the preservation of bone mineral density in postmenopausal women: a meta‐analysis. Osteoporos Int. 2015;26(5):1605‐1618. doi:10.1007/s00198-015-3034-0 25603795

[102] Gianoudis J , Bailey CA , Ebeling PR , et al. Effects of a targeted multimodal exercise program incorporating high‐speed power training on falls and fracture risk factors in older adults: a community‐based randomized controlled trial. J Bone Miner Res. 2014;29(1):182‐191. doi:10.1002/jbmr.2014 23775701

[103] Huovinen V , Ivaska KK , Kiviranta R , et al. Bone mineral density is increased after a 16‐week resistance training intervention in elderly women with decreased muscle strength. Eur J Endocrinol. 2016;175(6):571‐582. 27634943 10.1530/EJE-16-0521

[104] McCarthy B , Casey D , Devane D , Murphy K , Murphy E , Lacasse Y . Pulmonary rehabilitation for chronic obstructive pulmonary disease. Cochrane Database Syst Rev. 2015;(2):Cd003793. doi:10.1002/14651858.CD003793.pub3 25705944 PMC10008021

[105] Reid WD , Yamabayashi C , Goodridge D , et al. Exercise prescription for hospitalized people with chronic obstructive pulmonary disease and comorbidities: a synthesis of systematic reviews. Int J Chron Obstruct Pulmon Dis. 2012;7:297‐320. doi:10.2147/COPD.S29750 22665994 PMC3363140

[106] Spruit MA , Singh SJ , Garvey C , et al. An official American Thoracic Society/European Respiratory Society statement: key concepts and advances in pulmonary rehabilitation. Am J Respir Crit Care Med. 2013;188(8):e13‐e64. doi:10.1164/rccm.201309-1634ST 24127811

[107] Li S , Xi C , Li L , et al. Comparisons of different vitamin D supplementation for prevention of osteoporotic fractures: a Bayesian network meta‐analysis and meta‐regression of randomised controlled trials. Int J Food Sci Nutr. 2021;72(4):518‐528. doi:10.1080/09637486.2020.1830264 33043722

[108] Lips P , Cashman KD , Lamberg‐Allardt C , et al. Current vitamin D status in European and Middle East countries and strategies to prevent vitamin D deficiency: a position statement of the European Calcified Tissue Society. Eur J Endocrinol. 2019;180(4):P23‐p54. doi:10.1530/EJE-18-0736 30721133

[109] Nuti R , Brandi ML , Checchia G , et al. Guidelines for the management of osteoporosis and fragility fractures. Intern Emerg Med. 2019;14(1):85‐102. doi:10.1007/s11739-018-1874-2 29948835 PMC6329834

[110] Brask‐Lindemann D , Eiken P , Eskildsen P , Abrahamsen B . Time trends for alendronate prescription practices in women with chronic obstructive pulmonary disease and women exposed to systemic glucocorticoids. Osteoporos Int. 2013;24(6):1891‐1897. doi:10.1007/s00198-012-2220-6 23152095

[111] Smith B , Laslett L , Pile K , et al. Randomized controlled trial of alendronate in airways disease and low bone mineral density. Chron Respir Dis. 2004;1(3):131‐137. doi:10.1191/1479972304cd025oa 16281654

[112] Mazokopakis E , Starakis I . Recommendations for diagnosis and management of osteoporosis in COPD men. ISRN Rheumatol. 2011;2011:901416. doi:10.5402/2011/901416 22389805 PMC3263743

[113] Orwoll E , Ettinger M , Weiss S , et al. Alendronate for the treatment of osteoporosis in men. N Engl J Med. 2000;343(9):604‐610. doi:10.1056/NEJM200008313430902 10979796

[114] NIH consensus development panel on osteoporosis prevention, diagnosis, and therapy, March 7‐29, 2000: highlights of the conference. South Med J. 2001;94(6):569‐573. doi:10.1097/00007611-200194060-00004 11440324

